# Independent regulation of gene expression level and noise by histone modifications

**DOI:** 10.1371/journal.pcbi.1005585

**Published:** 2017-06-30

**Authors:** Shaohuan Wu, Ke Li, Yingshu Li, Tong Zhao, Ting Li, Yu-Fei Yang, Wenfeng Qian

**Affiliations:** 1 State Key Laboratory of Plant Genomics, Institute of Genetics and Developmental Biology, Chinese Academy of Sciences, Beijing, China; 2 Key Laboratory of Genetic Network Biology, Institute of Genetics and Developmental Biology, Chinese Academy of Sciences, Beijing, China; 3 University of Chinese Academy of Sciences, Beijing, China; 4 State Key Laboratory of Molecular Developmental Biology, Institute of Genetics and Developmental Biology, Chinese Academy of Sciences, Beijing, China; 5 Institute of Microbiology, Chinese Academy of Sciences, Beijing, China; National Center for Biotechnology Information (NCBI), UNITED STATES

## Abstract

The inherent stochasticity generates substantial gene expression variation among isogenic cells under identical conditions, which is frequently referred to as gene expression noise or cell-to-cell expression variability. Similar to (average) expression level, expression noise is also subject to natural selection. Yet it has been observed that noise is negatively correlated with expression level, which manifests as a potential constraint for simultaneous optimization of both. Here, we studied expression noise in human embryonic cells with computational analysis on single-cell RNA-seq data and in yeast with flow cytometry experiments. We showed that this coupling is overcome, to a certain degree, by a histone modification strategy in multiple embryonic developmental stages in human, as well as in yeast. Importantly, this epigenetic strategy could fit into a burst-like gene expression model: promoter-localized histone modifications (such as H3K4 methylation) are associated with both burst size and burst frequency, which together influence expression level, while gene-body-localized ones (such as H3K79 methylation) are more associated with burst frequency, which influences both expression level and noise. We further knocked out the only “writer” of H3K79 methylation in yeast, and observed that expression noise is indeed increased. Consistently, dosage sensitive genes, such as genes in the Wnt signaling pathway, tend to be marked with gene-body-localized histone modifications, while stress responding genes, such as genes regulating autophagy, tend to be marked with promoter-localized ones. Our findings elucidate that the “division of labor” among histone modifications facilitates the independent regulation of expression level and noise, extend the “histone code” hypothesis to include expression noise, and shed light on the optimization of transcriptome in evolution.

## Author summary

Gene expression noise, or cell-to-cell expression variability, has been a topic of intense interest for more than a decade. The prevailing model of “burst-like transcription” mediated by the promoter transitions between on and off states explains the formation of noise in eukaryotes. Albeit widely accepted, the *cis*- elements that determine the burst frequency and burst size remain largely unknown. Here we systematically examined the relationship between transcriptional burst frequency/size and all major histone modifications in various cell types, including human embryonic cells, mouse embryonic stem cells, and yeast, and found that histone markers can be divided into two groups based on their associations with burst frequency/ size. Coincidently, promoter-localized histone markers are associated with both burst size and burst frequency whereas gene-body-localized ones are more associated with burst frequency. We further knocked out a gene that is responsible for “writing” a gene-body histone mark in yeast, and found that burst frequency is indeed reduced. Our findings reveal a new mechanism of transcriptional burst regulation and shed light on the simultaneous optimization of gene expression level and noise in evolution.

## Introduction

Gene expression fluctuates among isogenic cells under identical conditions, which is frequently referred to as gene expression noise or cell-to-cell expression variability [1–3]. Noise can be decomposed into intrinsic noise and extrinsic noise, according to its origins. The inherent stochasticity of biochemical reactions (for example, collision between transcription factors and promoters) and the chromatin transition between on and off states together result in intrinsic noise [4]. Intrinsic noise can be amplified in the gene regulatory network, leading to varying concentrations of gene expression machinery (for example, polymerase II and ribosomes) among isogenic cells, which reinforces the cell-to-cell variability in gene expression and forms the basis of extrinsic noise.

Similar to expression level, expression noise is of central importance in growth, development, and responding to environmental fluctuations [1–3,5–12]. Thus, it is subject to natural selection and fine-tuned according to the gene function [7,8,10,13]. For instance, essential genes and genes encoding protein complex subunits, the expression variation of which is predicted to reduce fitness of the organism and thus collectively termed as dosage sensitive genes, often exhibit low expression noise [7,11,13]. By contrast, genes responding to environmental fluctuations are often associated with high expression noise, which could be a product of positive selection due to immediate or long-term benefits on fitness [6,10]. To summarize, expression level and noise are two facets of gene expression, both of which are subject to natural selection.

However, a strong negative correlation between expression level and noise has been observed in bacteria, yeast, and mammals [1,5,11,14–16]. For example, in *Saccharomyces cerevisiae*, Newman *et al* took advantage of the green fluorescent protein (GFP) collection in which each strain has a GFP fused to the C terminus of an endogenous protein, measured single cell protein levels in the cell population of each strain by fluorescence-activated cell scanning (FACS), and calculated expression level and noise for ~2000 genes. A strong negative correlation between expression level and noise was reported [11]. This negative correlation manifests as a potential constraint for simultaneous optimization of expression level and noise [17].

In yeast, a number of *cis-* acting elements have been suggested to regulate noise independent of expression level. For example, TATA-box containing genes have higher noise than TATA-less genes after controlling for expression level, suggesting its potential role in regulating gene expression noise [3,6,11,15,18]. Further, mutations in TATA-box result in marked decrease of both expression level and expression noise, suggesting the co-occurrence of high expression level and high noise enabled by TATA-box [14]. Another element is the sequence that determines the nucleosome occupancy around the transcriptional starting site (TSS). Occupied proximal-nucleosome (OPN) genes tend to have higher noise, while depleted proximal-nucleosome (DPN) genes tend to have lower noise, even though they display no significant difference in expression level [11,17,19]. In fact, adding nucleosome-disfavoring sequences and strengthening transcription factor binding sites exhibit different impacts on expression noise, even though they both elevate expression level [18]. Besides, a recent study shows that adaptive changes in the expression mean and noise of a gene with autoregulation occurred during the long-term evolution of yeast, suggesting that feedback is an alternative mechanism to decouple gene expression mean and noise [20].

In addition, epigenetic modification, which is closely associated with chromatin remodeling, has been suggested to play a role in regulating gene expression noise [21–26]. For example, Weinberger *et al* discovered that two histone deacetylation complexes, Set3 and Rpd3(L)C, play different roles in regulating gene expression noise although both repress expression level [27], implicating the potentially sophisticated regulation of transcription by histone modifications. However, the genomic landscape of all major histone modifications’ impacts on expression noise is yet to be reported. Here, we systematically investigated gene expression noise and discovered that histone modifications are associated with independent regulation of expression level and noise.

## Results

### Expression level and noise are negatively correlated in human embryonic cells

**Fig 1:**
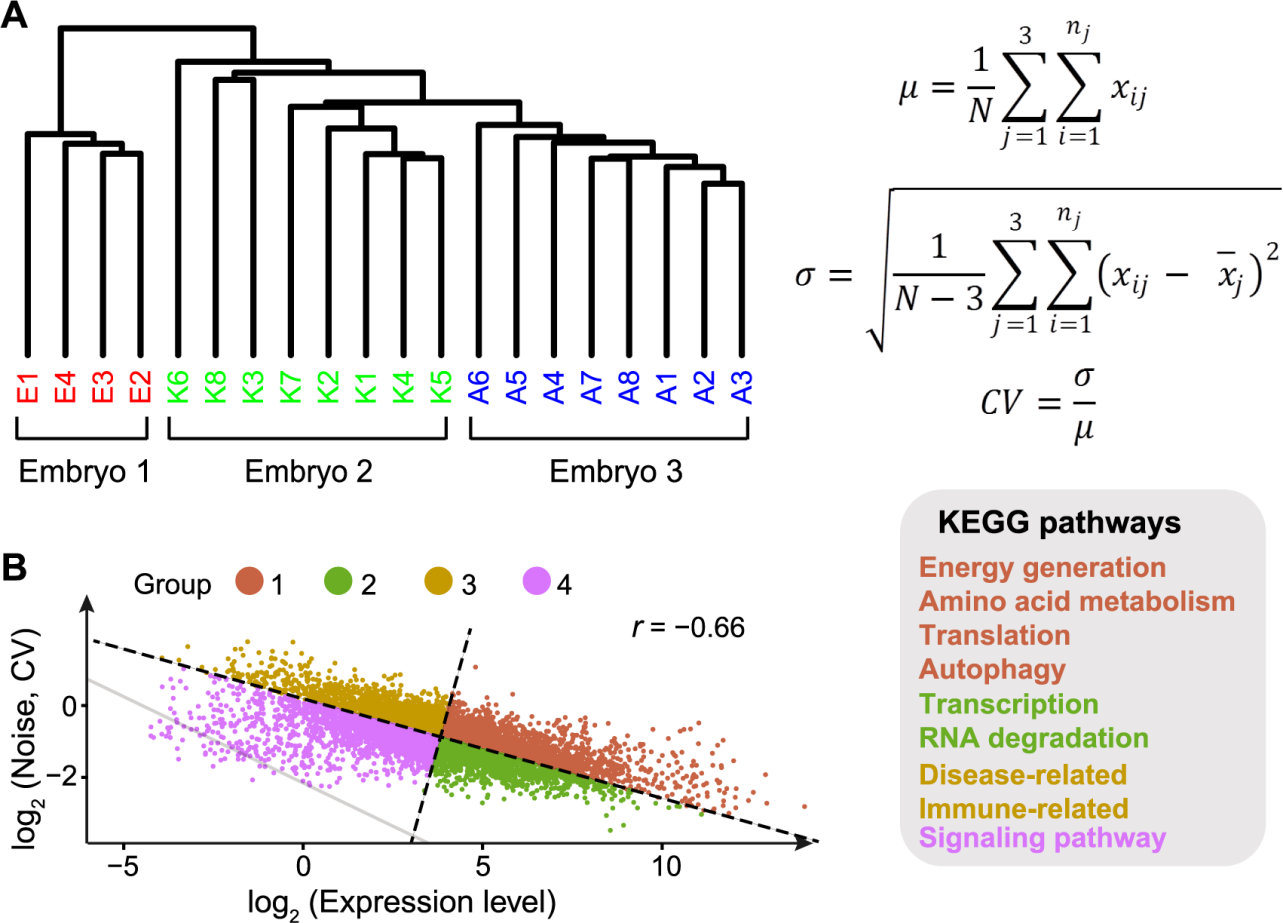
Functional enrichment among genes with different expression level and noise. **A**, **Left panel**: Unsupervised hierarchical clustering of the single-cell transcriptomes of 20 cells. 7741 genes with at least one sequencing read detected in all 20 cells were used for the analysis. **Right panel**: The formulae used to calculate expression level (*μ*) and noise (CV). **B**, **Left panel**: Expression noise is negatively correlated with expression level in human embryonic cells (*r* = -0.66, *P* < 1×10^−100^, *N* = 7741, Pearson’s correlation). Genes were further divided into 4 groups by the major axis and minor axis. Grey line indicates the estimated technical noise under given expression levels. **Right panel**: Enriched KEGG pathways in each group of genes.

Although gene expression noise has been extensively studied in single-celled organisms [1,5,11,14,15,28], noise in mammals, especially in human, has only been rarely addressed, either genome-wide [16,29] or on a small scale [30–32]. Single-cell mRNA-seq was performed in human preimplantation embryos [33], which provides us with the opportunity to investigate gene expression noise in human. Here, the expression noise of a gene is defined as the coefficient of variation (CV, *σ*/*μ*) of transcript concentrations among isogenic cells. We chose to use the data from 8-cell stage embryos for two reasons. First, the total number of single cells at this stage (20 cells from 3 embryos) is larger than that at 2- and 4-cell stages (6 and 12 cells, respectively). Second, it’s suggested that embryonic differentiation has not started yet at 8-cell stage [34]. To minimize the impact of sampling error during library preparation, which is larger for lowly expressed genes [35], we focused on the 7741 genes with at least one sequencing read detected in all 20 cells. With these data, we first performed unsupervised hierarchical clustering analysis, and found that cells from the same embryo do not always cluster together (Fig **1A**), suggesting that gene expression pattern in early embryos may not be fully determined by embryo identities. Consistently, the relatively long external branches compared to internal branches (Fig **1A**) indicate that these cells show unique gene expression patterns that are largely independent of their embryo identities. Nevertheless, to fully exclude the embryo effect in estimating expression noise, we subtracted the average expression of a gene in cells of the same embryo from its expression when calculating gene expression noise (Fig **1A**). Consistent with previous findings in yeast [4,11,14,15] and in a mouse cell line[36], we observed a strong negative correlation between expression level and noise in human embryonic cells (Fig **1B**, *r* = -0.66, *P* < 1×10^−100^, *N* = 7741, Pearson’s correlation). Note that for most genes, the noise estimated from single-cell mRNA-seq data is higher than technical noise (indicated by the grey line in Fig **1B**), suggesting that technical noise is not the main source of the observed correlation above.

It is worth noting that the observed correlation between expression level and noise is potentially a constraint that prevents simultaneous optimization of both. Nevertheless, we observed that many genes, low-expression-low-noise and high-expression-high-noise genes of particular interest, deviated from the regression line in the expression level-noise correlation (Fig **1B**). To examine whether this is related to biological functions, we divided genes into four groups based on their expression level and noise, and found that genes in these groups are enriched in various biological pathways (Fig **1B** and **S1** Table). More importantly, an inspection of these pathways reveals that the observations are consistent with the direction of natural selection on expression level and noise. For instance, genes in signaling transduction pathways, which generally function through the accurate expression of minute amounts of specific products, are enriched in the-low-expression-low-noise group (Group 4, Fig **1B**). On the contrary, genes in the pathways of energy generation, amino acid metabolism, and autophagy, which benefit the organism by sensing and responding to environmental fluctuations, usually demand both high expression and high noise [6,10,37]. Consistently, they are enriched in the high-expression-high-noise group (Group 1, Fig **1B**). We further defined genes with significant deviation as those deviated from the 95% confidence intervals of the major axis and minor axis, and reanalyzed the enriched pathways in 4 groups of genes, which generated similar results (**S2** Table). More generally, we calculated the ratio between the numbers of essential and nonessential genes in each group, and found that essential genes are significantly enriched in the high-expression-low-noise group (Group 2, **S1A** Fig). Similar result was obtained for genes encoding protein complex subunits (Group 2, **S1B** Fig). We further verified that the observed pattern is not an artifact of varying mRNA decay rates among human genes, because mRNA decay rate [38]is only weakly correlated with expression level and noise (**S2** Fig). Taken together, we found that regardless of the strong correlation between expression level and noise, the decoupling of them is prevalent in human embryonic cells.

### Decoupling between gene expression level and noise is potentially through separate modulation of transcriptional burst frequency and burst size by histone modifications

**Fig 2:**
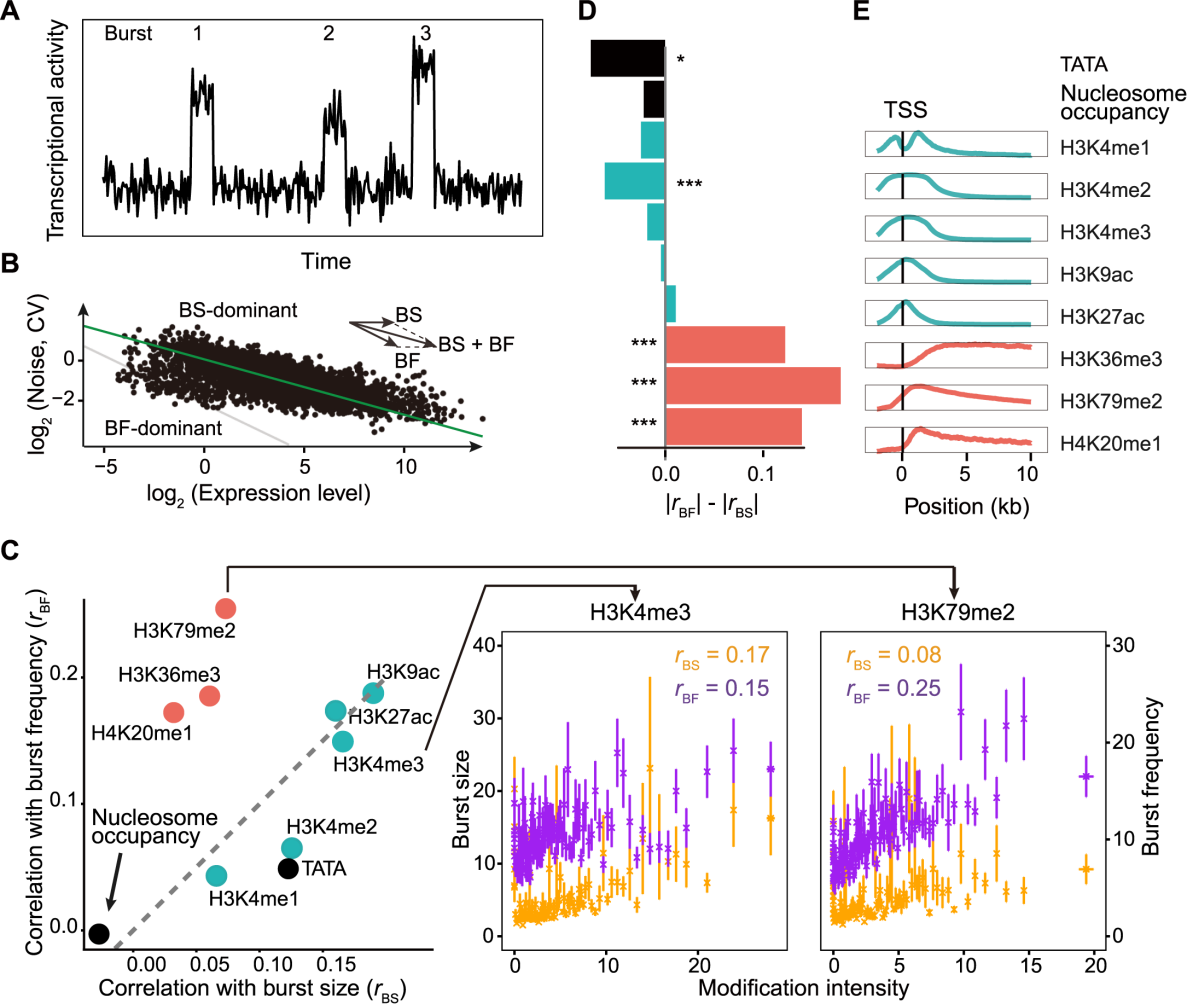
Separate association with burst frequency and size among histone modifications. **A**, The burst-like gene expression model. **B**, Similar to Fig **1B**, expression level and noise of each gene in the human genome were plotted, where each dot represents one gene. Burst-size-dominant genes are above the major axis, while burst-frequency-dominant genes are below the major axis. **C**, **Left panel:** Correlations between the intensity of each histone modification and burst frequency and burst size (*r*_BF_ and *r*_BS_, respectively) were shown, as well as *r*_BF_ and *r*_BS_ for the status of TATA box and nucleosome occupancy. **Right panel:** Correlations between the intensity of H3K4me3/H3K79me2 and burst size/frequency, respectively, as an illustration of the correlation calculation. Genes were divided into 200 equal-sized bins (bins with equal number of genes) based on their H3K4me3 or H3K79me3 intensity. In each bin of genes, the mean and standard error of H3K4me3 (or H3K79me2) intensity and burst size/frequency were calculated. In the last several bins, the variation of H3K4me3 (or H3K79me2) intensity is relatively larger, so the error bars are broader. Correlation coefficients were calculated with the raw data (*N* = 3350). **D**, The difference of the absolute values of *r*_BF_ and *r*_BS_. Permutation test was used to determine the significance of difference between *r*_BF_ and *r*_BS_. *N* = 3350. ***, *P* < 0.001; **, *P* < 0.01; *, *P* < 0.05. **E**, Average distributions of 8 histone modifications in human embryonic cells, aligned by TSS. Promoter-localized and gene-body-localized histone modifications are marked in cyan and coral, respectively.

We next turned to the transcriptional process for a molecular interpretation of the decoupling. It is generally accepted that eukaryotes, the DNA of which is wrapped around histones forming nucleosomes, mainly adopt a burst-like transcription process [2–4,9,14,17,29,39], due to the chromatin remodeling mediated promoter on-and-off transitions (Fig **2A**). It was previously observed that a large fraction of genes in human adopt the burst-like expression mode [29,40]. In this prevailing “burst model”, when the promoter is “on”, a number of mRNA are transcribed, which is called a burst event. The frequency of such burst events is defined as burst frequency and the average number of mRNA molecules made per event is defined as burst size. Therefore, expression level changes with both burst frequency and burst size, while expression noise changes mainly with burst frequency (Fig **2B** & **S3** Fig) [14,17]. Thus, if expression level is predominantly regulated by burst size at the genomic scale, the slope of the regression line between expression level (*μ*) and noise (standard deviation divided by mean, CV, *σ/μ*) should be approximately 0 (Fig **2B**). By contrast, if expression level is predominantly regulated by burst frequency, the slope should be approximately -0.5, because CV is inversely proportional to the square root of mean in a Poisson process (Fig **2B**). In Fig **2B**, the slope falls between 0 and -0.5, suggesting that both burst size and burst frequency are modulated across the human genome. Meanwhile, deviation from the regression line suggests that the relative contributions of burst frequency and burst size vary among genes. Specifically, genes above the regression line have relatively larger burst size while those under the line have relatively larger burst frequency, given similar expression levels. For instance, genes in Groups 1 and 3 have relatively larger burst size but smaller burst frequency than genes in Groups 2 and 4, respectively (Fig **1B**). Based on this, we hypothesized that the decoupling of expression level and noise is enabled by the separate modulation of burst frequency and size.

Therefore, we estimated burst size and burst frequency of transcription for each gene from the single-cell mRNA-seq data [33] following previous studies [29,41]. mRNA level measured by mRNA-seq is proportional but not necessarily equal to the mRNA number of a gene in a cell, due to the amplification during mRNA-seq library preparation and high throughput sequencing. Because this amplification influences the estimation of the Fano factor and thus burst size, we first calibrated the mRNA number of each gene in a cell by the total number of mRNA in a typical mammalian cell (see Methods and Materials for details) [42]. We then estimated burst size with the equation *burst size* = *σ*^2^/*μ* -1, in which *σ*^2^/*μ* is the Fano factor. We further calculated burst frequency by calculating the ratio between the mRNA number of a gene in a cell and the estimated burst size (see Methods and Materials).

Epigenetic modification, especially histone modification, has been reported to play vital roles in regulating expression noise [27]. To systematically examine the roles histone modifications play in transcriptional regulation, we first retrieved the genomic distribution data of all the major euchromatic histone modifications, H3K4me1, H3K4me2, H3K4me3, H3K9ac, and H3K27ac, H3K36me3, H3K79me2, and H4K20me1, which were obtained from the chromatin immunoprecipitation sequencing experiments (ChIP-seq) conducted in human embryonic stem cells (hESC), from the ENCODE project [43]. Specifically, we defined 2000 base pairs upstream of TSS to transcription end site (TES) as the range of a gene and obtained the called ChIP-seq peaks for each histone mark in this range. In this study, the strength of a histone modification on a gene was defined as the average intensity of this histone mark on the gene, which was calculated as the ratio between the total intensity (total “height” of all peaks) of a histone mark on a gene and the range of the gene (see Methods and Materials for details).

We calculated the Pearson’s correlation between the intensity of each histone modification and transcriptional burst frequency/size. Surprisingly, we found that these histone modifications can be divided into two distinct groups based on the correlations with burst frequency and size. Specifically, three histone modifications (H3K36me3, H3K79me2, and H4K20me1) have significantly stronger correlations with burst frequency (*r*_*BF*_) than with burst size (*r*_*BS*_), while other histone modifications in general exhibit little differences between *r*_*BF*_ and *r*_*BS*_ (Fig **2C and 2D**, significance determined by permutation test). Importantly, this distinction coincides with that according to the localization of histone modifications (Fig **2E**). That is, gene-body-localized histone modifications exhibit larger *r*_*BF*_, while promoter-localized ones (including the enhancer-localized marker H3K4me1) often show similar *r*_*BF*_ and *r*_*BS*_. It is important to note that mRNA decay rate was not included in the estimation of burst parameters here, due to the lack of such data in human embryonic cells. Therefore, we used the approximation that the mRNA degradation rate is identical among transcripts and set this rate to 1 min^-1^. Nevertheless, when we used the mRNA decay rate obtained from human lymphoblastoid cell lines as a substitute [38], the observation in Fig **2** persisted (**S4** Fig).

**Fig 3:**
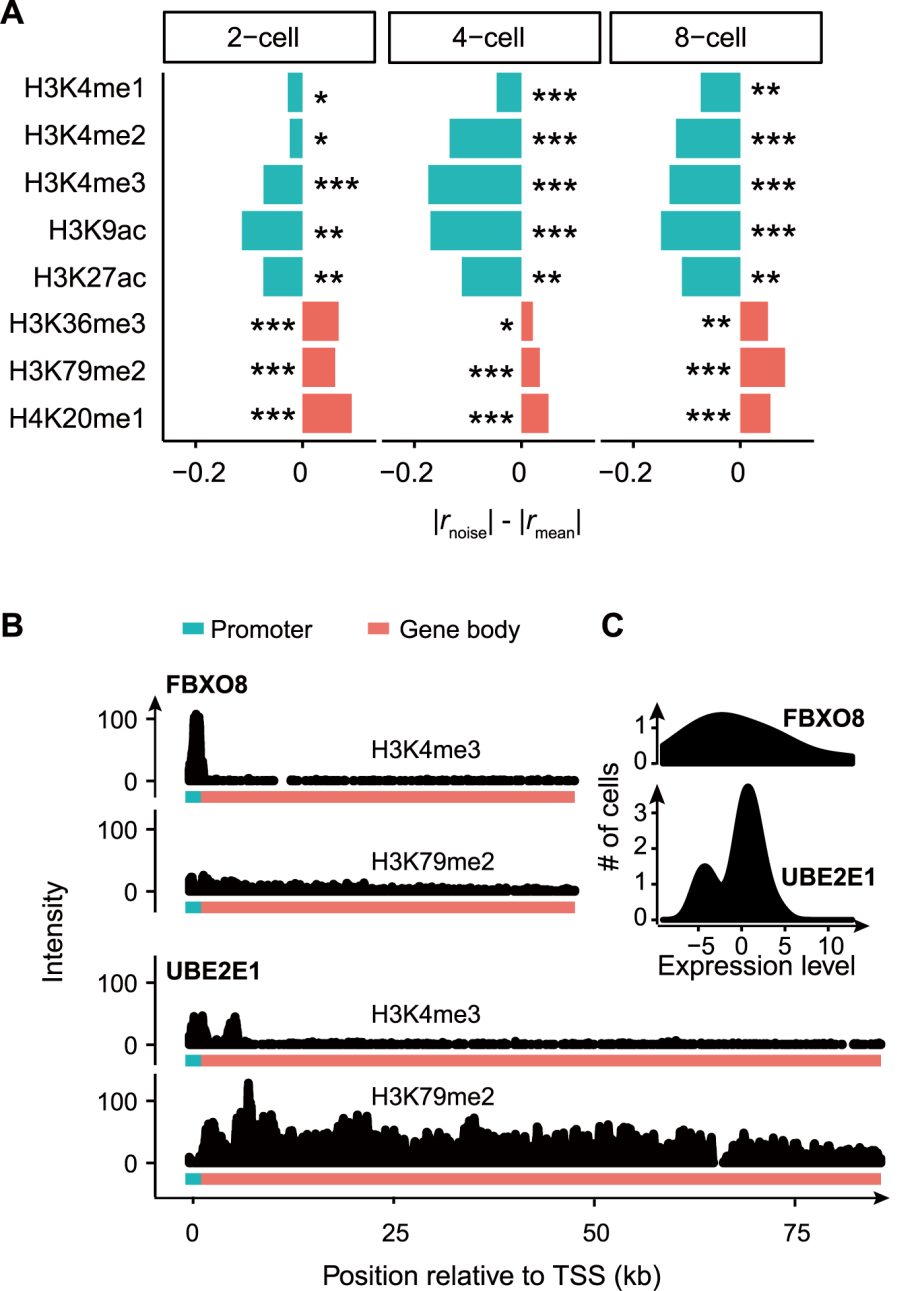
Decoupling of expression level and noise enabled by histone modifications in human embryonic cells. **A**, The difference of the absolute values of *r*_noise_ and *r*_mean_ in 2-, 4-, and 8-cell stages of human preimplantation embryos. Permutation test was used to determine the significance of difference between *r*_noise_ and *r*_mean_. *N* = 6731, 8131, and 7015 for 2-cell, 4-cell, and 8-cell stage, respectively. **B**, The gene encoding F-box protein 8 (*FBXO8*) is intensively marked by H3K4me3 in promoter and weakly marked by H3K79me2 in gene body, while the gene encoding ubiquitin-conjugating enzyme E2E 1 (*UBE2E1*) shows an opposite pattern. The region of 2000bp upstream of TSS is shown as the promoter and the region between TSS and TES is shown as the gene body. **C**, The distributions of expression levels of *FBXO8* and *UBE2E1* in 20 cells are shown, respectively. Smoothed density estimates were displayed.

Since expression level is jointly determined by burst frequency and burst size, while expression noise is mainly determined by burst frequency, we predict that promoter-localized histone modifications are more strongly associated with expression level (|*r*_mean_| > |*r*_noise_|) while gene-body-localized ones are more strongly associated with expression noise (|*r*_noise_| > |*r*_mean_|), which was indeed observed in 2-cell, 4-cell, and 8-cell embryos (Fig **3A**). This suggests that the independent regulation of expression level and noise may be enabled through separate modulation of burst size and burst frequency by histone modifications. As an example, the low-noise gene *UBE2E1* is intensively modified by the gene-body-localized histone mark H3K79me2 but only sparsely modified by the promoter-localized histone mark H3K4me3, while the high-noise gene *FBXO8* displays the opposite pattern (Fig **3B and 3C**).

It is worth noting that expression level and expression noise were estimated in embryonic cells whereas histone modification data are from hESC, due to the lack of such data in human embryonic cells. To test whether this can lead to artifacts in our analysis, we further examined histone modification conservation between two substantially different cell types, hESC and GM12878 (a lymphoblastoid cell line), in ENCODE. We found that the intensity of each histone mark is highly correlated between these two cell types (Spearman’s correlation coefficient *ρ* ranges from 0.46 to 0.83, **S5** Fig), suggesting that histone modification state may be largely similar across cell types. More importantly, we calculated correlation between the intensities of histone modifications in GM12878 and the expression level (noise) in cells from the 8-cell stage embryos. The pattern observed in Fig **3** is largely unchanged (**S6** Fig), suggesting that our observation is not sensitive to the cell type in which histone modification was quantified.

To summarize, histone modifications in human embryonic cells can be divided into two distinct groups based on their associations with burst frequency and size. Although the impact of individual histone modification seems modest, the combined effect of multiple markers on the same gene could be strong. It is worth noting that the association between gene-body-localized histone markers and burst frequency is not likely the consequence of transcriptional elongation mediated by these markers [44–46], because transcriptional elongation is unlikely to determine transcriptional burst frequency (but see [22]).

### Histone modification is associated with the decoupling between gene expression level and noise in yeast

**Fig 4:**
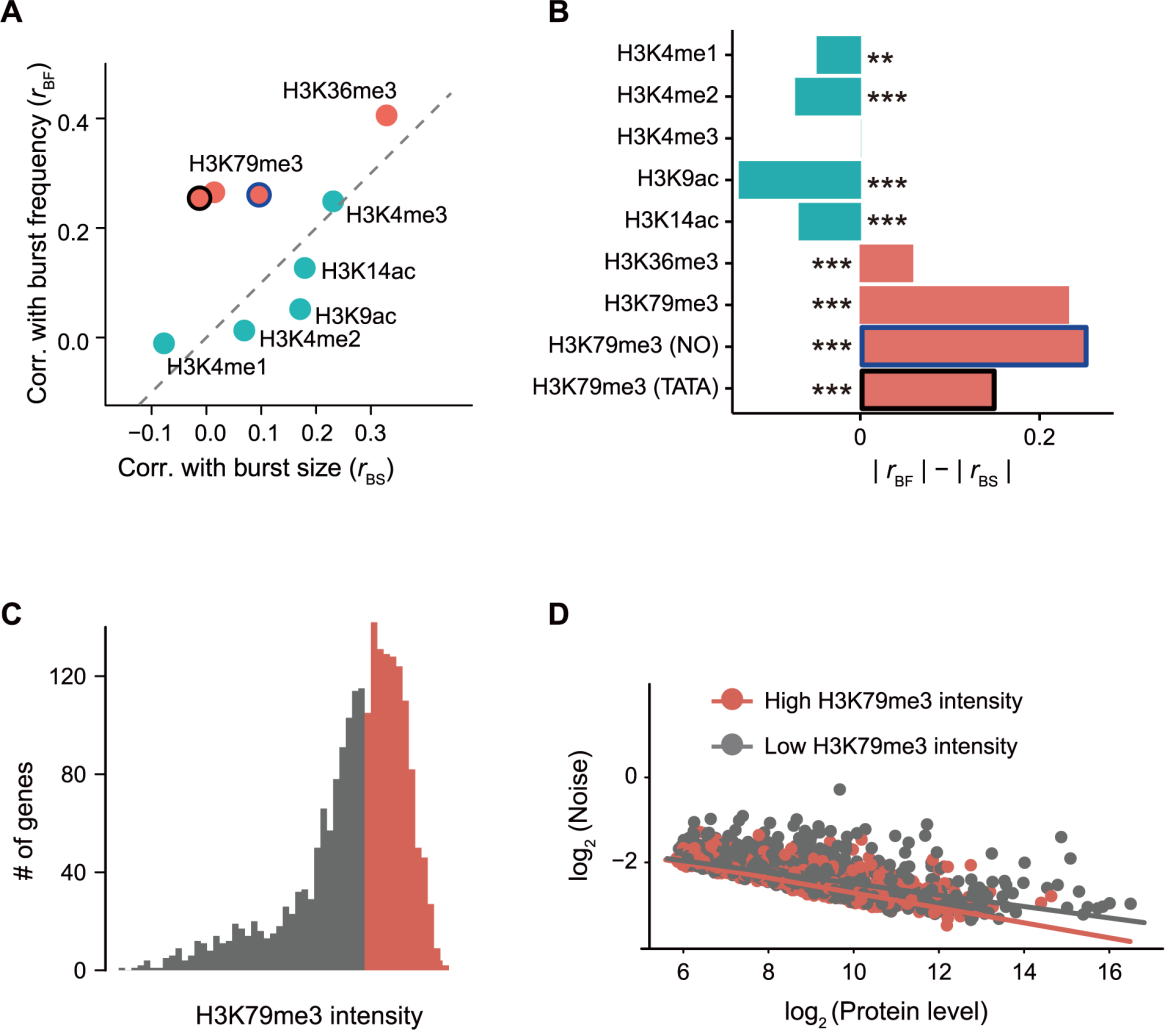
Decoupling of expression level and noise in yeast. **A**, Correlations between the intensity of each histone modification and burst size/burst frequency. **B**, The difference of the absolute values of *r*_BF_ and *r*_BS_ in yeast. Permutation test was used to determine the significance of difference between *r*_BF_ and *r*_BS_. *N* = 2168 for the first 7 bars, *N* = 468 for H3K79me3 (Nucleosome occupancy), and *N* = 2163 for H3K79me3 (TATA), respectively. ***, *P* < 0.001; **, *P* < 0.01; *, *P* < 0.05. Spearman’s correlation coefficients were calculated in **A** and **B**, because the logarithm of expression levels among genes do not fit into normal distribution in yeast (Shaprio test of normality, *P* = 4.1×10^−33^). **C**, Genes are divided into 2 equal-sized groups based on H3K79me3 intensity in the wild-type strain. **D**, Genes with high-intensity H3K79me3 have larger burst frequency, which is illustrated by the steeper slope of the regression line (regression coefficients: -0.18 for genes with high-intensity H3K79me3 vs. -0.13 for genes with low-intensity H3K79me3, linear regression, *P* = 3.5×10^−12^, *df* = 2163).

The budding yeast *Saccharomyces cerevisiae* has been frequently used to study gene expression noise [2,5,6,8,11,14,15]. With the yeast GFP collection in which the coding sequence of GFP is fused to the C-terminus of an endogenous gene in each strain, Newman et al (2006) measured single-cell fluorescence of ~2000 strains with FACS [11]. With these data, we next investigated the association of histone modifications [47] with expression level and noise in yeast. We calculated burst frequency and burst size of these genes and calculated correlation coefficients between them and the intensity of each histone modification. Consistent with the findings in human embryonic cells, we found that *r*_*BF*_ is significantly larger than *r*_*BS*_ for the gene-body-localized histone modifications, such as H3K79me3 (*r*_*BF*_ = 0.26, *r*_*BS*_ = 0.03, permutation test *P* value < 0.001, Fig 4**A and** 4**B**), while promoter-localized histone modifications tend to have similar *r*_*BS*_ and *r*_*BF*_. Consistent with this, genes with high H3K79me3 intensities have a steeper slope than those with low H3K79me3 intensities (Fig 4**C and** 4**D,** linear regression *P* = 3.5×10^−12^, *df* = 2163), which indicates that burst frequency is modulated to a larger extent among genes with high H3K79me3 intensities, suggesting the role of H3K79me3 in preferential modulation of burst frequency. The consistent pattern in multiple human embryonic stages (Fig **3A**), in yeast (Fig **4**), and in mouse embryonic stem cells (see below) suggests that the role of histone modifications in decoupling expression level and noise is evolutionarily conserved.

Since TATA box and nucleosome occupancy are also suggested to play a role in the decoupling of expression level and noise in yeast [3,6,11,15,18,19], we calculated partial correlations between H3K79me3 intensity and gene expression noise controlling for these two factors. It turns out that the correlation coefficients stay virtually unchanged (Fig **4A and** 4**B**), suggesting that H3K79me3 is associated with noise through a TATA or nucleosome occupancy independent mechanism.

### H3K79 methylation plays a causal role in repressing gene expression noise in yeast

**Fig 5:**
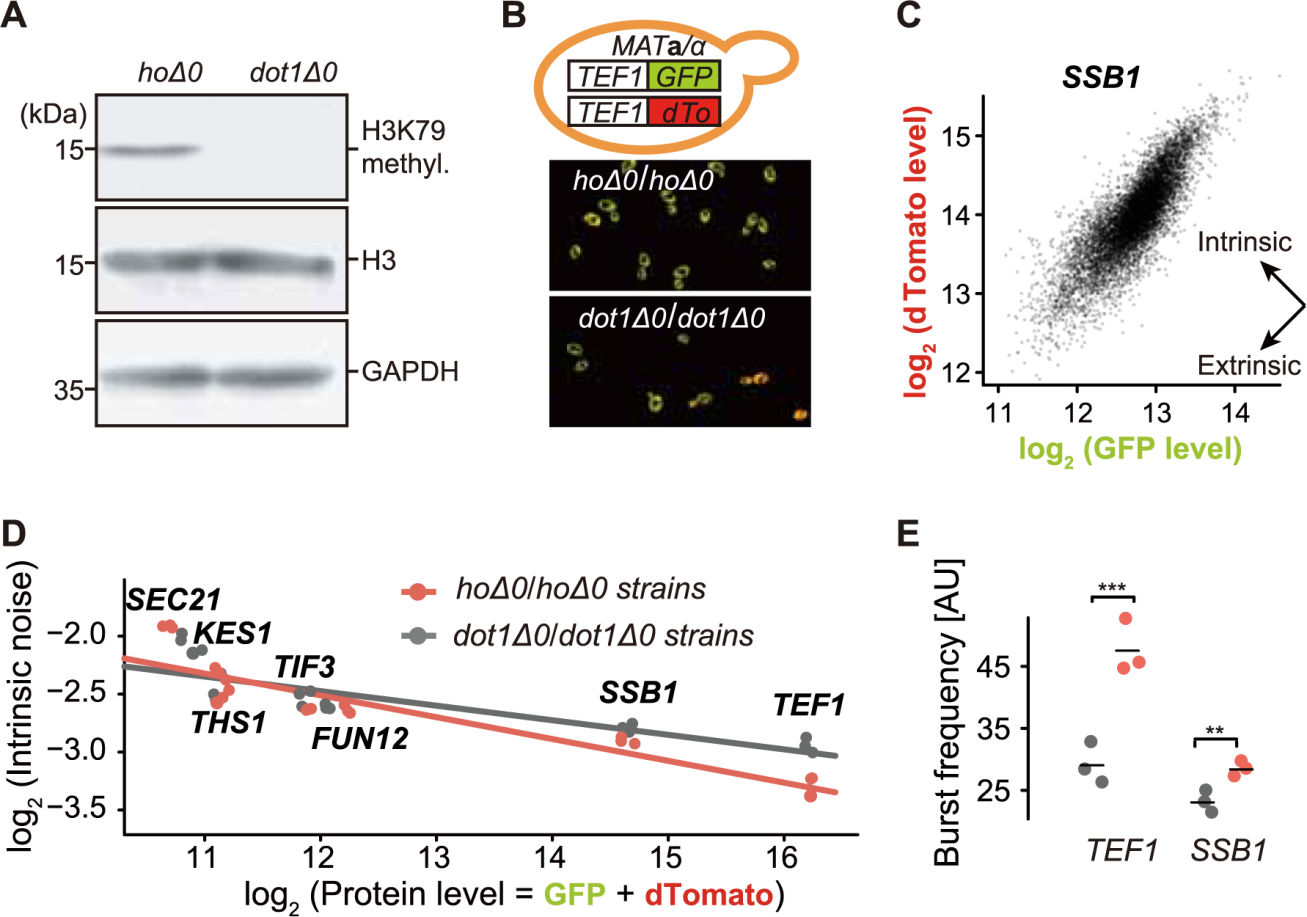
H3K79 methylation plays a causal role in repressing gene expression noise in yeast. **A**, The absence of H3K79 methylation in *DOT1* deletion strains was confirmed with western blot. **B**, Yeast two-color system for measuring intrinsic noise and on *TEF1*. **C**, The fluorescence intensities of GFP and dTomato among single cells were measured by FACS. Fluorescence levels are in arbitrary units. **D**, *DOT1* deletion flattens the noise-protein level regression (regression coefficients: -0.19 in the *HO* deletion background vs. -0.13 in the *DOT1* deletion background, linear regression, *P* = 0.03). **E**, Burst frequencies of *TEF1* and *SSB1* are reduced in the *DOT1* deletion strain. ***, *P* < 0.005; **, *P* < 0.01.

Our observations so far are mainly from correlation analyses. We next designed experiments to examine the causality between histone modifications and expression level/noise. Here, we used H3K79 methylation as an example, because it has only one “writer” (*DOT1*, a non-essential gene) in yeast [46], which makes the removal of H3K79 methylation more feasible. Specifically, we constructed a homozygous *DOT1* knockout strain and confirmed the absence of H3K79 methylation with western blot (Fig **5A**). A pseudo gene (*HO*) homozygous knockout strain was similarly constructed as a negative control. To examine whether intrinsic noise level is elevated in the *DOT1* knockout strain, a two-color system was constructed following previous studies [1,3], in which *GFP* and a red fluorescent protein gene (*dTomato*) were respectively fused to two alleles of the same endogenous gene (Fig **5B**). Since two fluorescent proteins are expressed from the same promoter in the same cellular environment, the fluorescence difference between them is only attributable to intrinsic noise. We first examined intrinsic noise of *TEF1*, which is reported to be extensively modified by H3K79 methylation on its gene body [47], and observed that the fluorescence difference between GFP and dTomato is larger in *DOT1* knockout strain (Fig **5B**), suggesting that removal of H3K79 methylation elevates the intrinsic noise of *TEF1*.

To further quantify intrinsic noise, we randomly chose 6 additional genes with different H3K79 methylation intensities in wild-type cells, as reporter genes (**S3** Table), and measured their fluorescence intensities of GFP and dTomato with FACS (Fig **5C**). We calculated intrinsic noise in *DOT1* and *HO* knockout strains following previous studies [1,3], and found that *DOT1* deletion indeed elevates expression noise, especially for highly expressed genes (*SSB1* and *TEF1*, Fig **5D**). Importantly, the slope difference between regression lines in *HO* and *DOT1* knockout strains (Fig **5D**) is consistent with that among genes with high and low H3K79me3 intensities (Fig **4D**). In further support of our model, both *SSB1* and *TEF1* exhibit lower burst frequencies in *DOT1* deletion strains (Fig **5E**). These observations are consistent with a recent genome-scale study about the position effects on gene expression noise [25]. In that study, Chen and Zhang reported that genes inserted into genomic regions with high H3K79me3 tended to have lower expression noise in yeast. To summarize, H3K79 methylation plays a causal role in repressing gene expression noise, which supports our hypothesis that independent regulation of expression level and noise is enabled by histone modifications.

Unexpectedly, we also observed that expression level virtually does not change upon *DOT1* deletion, potentially due to some compensatory change of burst size accompanying the decrease of burst frequency. Alternatively, the compensation may occur at the post-transcriptional level. For example, protein degradation rate may change upon dosage imbalance among genes [48], so the altered protein degradation rate could lead to the compensation of protein concentration in *DOT1* deletion strains. Importantly, the observation of compensation in our experiment is consistent with the evolutionary change of gene expression between human and mouse (see Discussion).

### Histone-modification-associated independent regulation of expression level and noise

**Fig 6:**
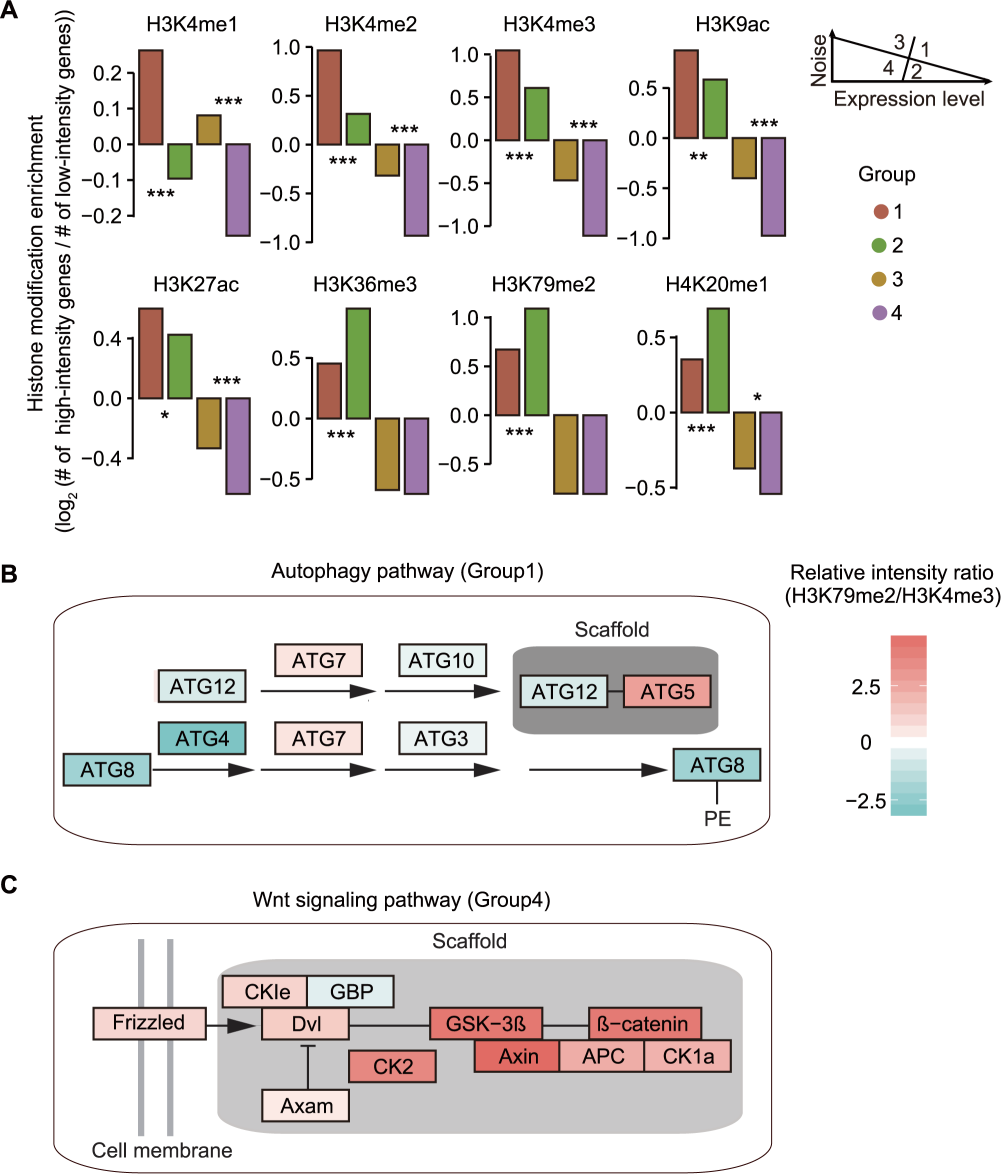
Usage preference of histone modifications. **A**, Genes intensely modified by promoter-localized histone markers are enriched in high-noise groups (1 and 3), while genes intensely modified by gene-body-localized histone markers are enriched in the low-noise Group 2. Hypergeometric test was used to determine the enrichment significances. **B**, Genes regulating autophagy have lower relative H3K79me2/H3K4me3 intensity ratio. **C**, Genes in the Wnt signaling pathway have higher relative H3K79me2/H3K4me3 intensity ratio.

Genes with divergent functions have unique combinations of expression level and noise (Fig **1B**). Are such unique combinations enabled by the histone coding strategy described above? To address this question, we first examined the enrichment of histone modifications in each group of genes defined in Fig **1B**. Specifically, for each histone modification, we classified genes into high-intensity ones and low-intensity ones with the median, and calculated the ratio between the numbers of them in each group, which reflects the usage preference of this histone modification among genes in the group. We found that genes with both high expression level and high noise (Group 1) are preferentially modified by promoter-localized histone markers, while genes with high expression level and low noise (Group 2) are preferentially modified by gene-body-localized ones in human (Fig **6A**). We further illustrated this point with two pathways, autophagy pathway in Group 1 and Wnt signaling pathway in Group 4. Genes in autophagy pathway respond to intracellular and extracellular stimuli, and thus, are predicted to have higher noise. Indeed, we found that they are preferentially modified by the promoter-localized histone marker H3K4me3 (Fig **6B**). By contrast, genes in Wnt signaling pathway require low noise to ensure the accurate expression of minute amounts of their products, thus guaranteeing the fidelity in signaling transduction. Consistently, these genes are preferentially modified by the gene-body-localized histone marker H3K79me2 (Fig **6C**). Additional examples (oxidative phosphorylation signaling pathway in Group1 and Jak-STAT signaling pathway in Group4) are shown in **S7** Fig. We then performed a similar enrichment analysis in yeast and observed virtually the same pattern as in human embryonic cells (**S8** Fig). Interestingly, H3K36me3 is enriched among Group 4 genes but not Group 2 genes, suggesting that H3K36me3 may play different roles in highly expressed and lowly expressed genes in yeast.

**Fig 7:**
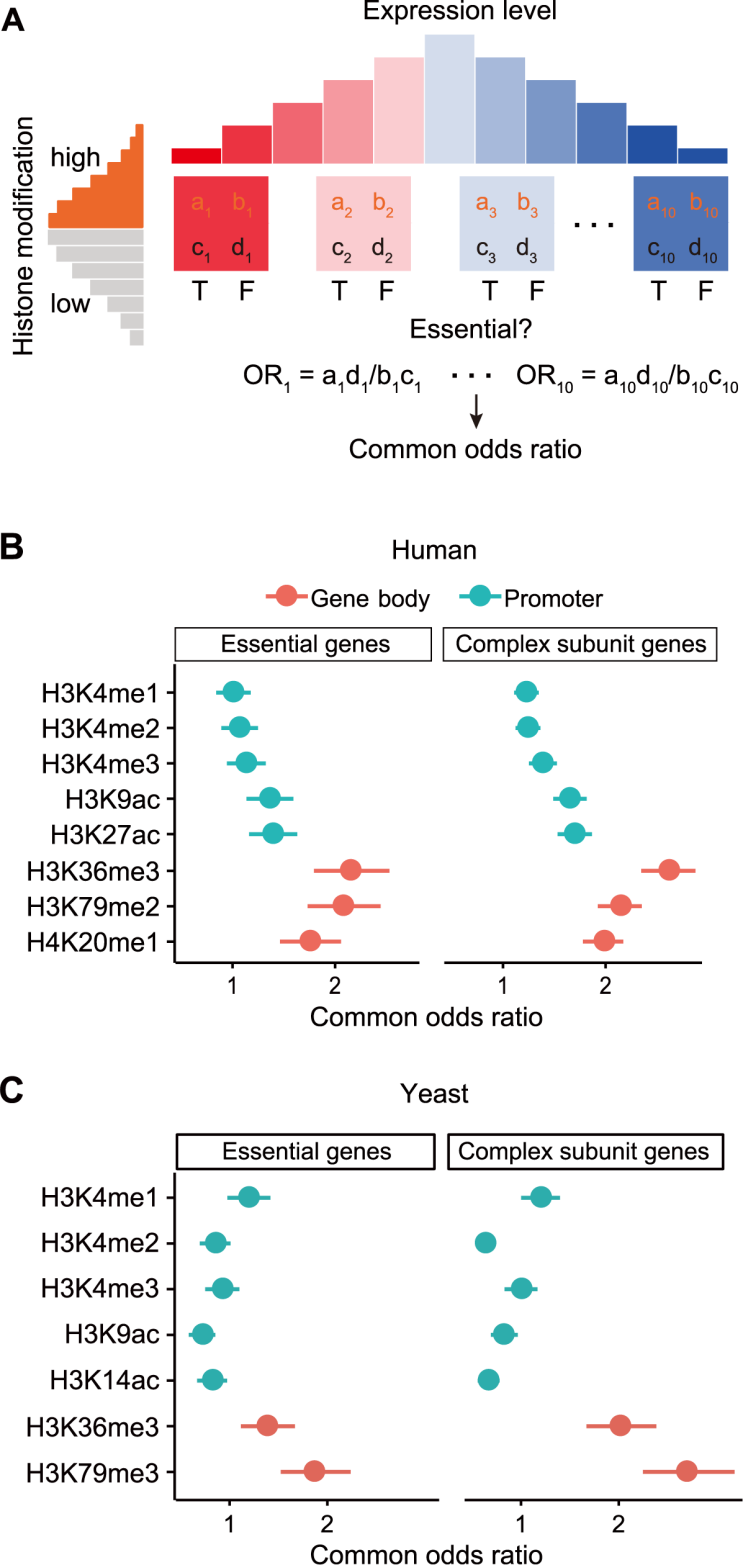
Dosage sensitive genes are preferentially modified by gene-body-localized histone markers. **A**, Calculation of histone modification usage preference after controlling for expression level. **B-C**, Dosage sensitive genes are preferentially modified by gene-body-localized histone markers both in human embryonic cells (**B**) and yeast (**C**). Error bars represent the 95% confidence intervals of the common odds ratios.

More broadly, dosage sensitive genes (e.g., essential genes and genes encoding protein complex subunits) tend to have lower noise both in yeast [7,11] and in human (**S1** Fig). To test if these genes are preferentially modified by gene-body-localized histone markers, we calculated usage preference of each histone modification among them. To exclude the confounding effect of expression level, we first divided genes into 10 equal-sized bins based on expression level. Then in each bin, we divided genes into four categories based on the intensity of histone modifications and dosage sensitivity. With that, we calculated an odds ratio of the contingency table in each bin, and then calculated a common odds ratio and 95% confidence interval with Mantel-Haenszel procedure (Fig **7A**). Consistently, we found that dosage sensitive genes indeed prefer to use gene-body-localized histone modifications, which is reflected by the larger-than-one common odds ratios, in both human embryonic cells and yeast (Fig **7B and 7C**). All these observations indicate that histone modifications play a vital role in independent regulation of expression level and noise in human and yeast genomes.

## Discussion

### Negligible impact of cell differentiation at the 8-cell stage on noise calculation

In this study, we calculated expression noise among cells from three 8-cell stage embryos, considering that the fraction of maternal mRNA has declined to reach parity with paternal transcripts [49] and the number of single cells (20 cells) is relatively large. However, it is recently reported that a small proportion of genes display bimodal expression at 2- or 4- cell stage in mouse, implying potential cell differentiation in human 8-cell stage embryos [50], which may confound the calculation of expression noise. Nevertheless, we hold that the observations in this study are not artifacts, with the following supporting evidence. First, we calculated expression level and noise from human 2-cell stage (*N* = 6), 4-cell stage (*N* = 12), and embryonic stem cells (*N* = 30), and observed “decoupling” of expression level and noise in all these samples (Fig **3A** & **S9A** Fig). Second, we further excluded human homologs of the bimodally expressed genes in mouse at 2- or 4-cell stage [50] and obtained essentially the same pattern (**S9B** Fig). Third, similar pattern was also obtained in mouse embryonic stem cells (*N* = 94, **S9D** Fig), as well as in yeast (Figs **4 and 5**). The consistent patterns observed in all these samples imply the negligible contribution of potential differentiation at the 8-cell stage to noise calculation.

### The divergence of H3K79me2 intensity between human and mouse is associated with the divergence of gene expression noise

So far, we have observed that the intensities of gene-body-localized histone modifications are associated with expression noise among genes (Figs **2**–**4**). Next, we investigated whether the same pattern could be observed among orthologous genes between human and mouse. Specifically, we asked whether the change of H3K79me2 intensity on a gene in evolution could predict the divergence of expression noise. To this end, we obtained the H3K79me2 intensities as well as single-cell transcriptomes in embryonic stem cells of human and mouse [33,51]. We observed that the change of H3K79me2 intensity could successfully predict the divergence of expression noise between human and mouse (**S10** Fig, *ρ* = -0.12, *P* = 1.1×10^−6^), suggesting that the modulation of H3K79me2 intensity may play a role in the evolutionary optimization of gene expression noise. Interestingly, we did not detect a significant correlation between the change of H3K79me2 intensity and the divergence of expression level (**S10** Fig, *ρ* = -0.03, *P* = 0.2). This could be due to the same compensatory mechanisms underlying the pattern observed in our manipulative experiment in yeast (Fig **5**).

### Negligible function of TATA and nucleosome occupancy in regulating expression noise in human embryonic cells

In yeast, TATA box and TSS-proximal nucleosome occupancy are associated with high noise independent of expression level [3,6,11,14,15,18,19]. In our study, however, we found that in human embryonic cells, neither the presence/absence of TATA-box [52,53] (*ρ* = 0.05, *P* = 0.10, *N* = 1393) nor the TSS-proximal nucleosome occupancy [54] (*ρ* = -0.003, *P* = 0.88, *N* = 3350, Fig **2C**) is correlated with burst frequency.

Consequently, the difference in expression noise between TATA-box containing and TATA-less genes is not significant, and TSS-proximal nucleosome occupancy is only weakly correlated with noise (**S11** Fig). This result persists when we classified genes according to the presence of a canonical TATA-box (TATAAA, **S11A** Fig) and calculated nucleosome occupancy in various ranges (**S11B** Fig), which implies that alternative mechanisms should exist to overcome the constraint between expression level and noise in human embryonic cells. We speculated that the losses of functions of TATA-box and nucleosome occupancy in expression noise regulation are compensated by the role of histone modifications in mammals.

### Histone modifications may regulate expression noise through chromatin accessibility

Previous studies have provided some potential mechanisms by which chromatin structure and histone modifications regulate gene expression noise [3,6,11,15,18,19,21–26]. Weinberger *et al* discovered that histone deacetylase complex Rpd3(L)C could regulate expression noise by modulating transcription initiation [27]. Benayoun *et al* observed a correlation between H3K4me3 breadth and transcriptional consistency among single cells [22]. Benayoun *et al* further confirmed that the perturbation of H3K4me3 breadth led to reduced transcriptional consistency. They proposed that H3K4me3 breadth might regulate transcriptional consistency through a positive feedback loop between transcription initiation and elongation. However, in a genome-scale experiment that examined the position effects on gene expression noise in yeast [25], Chen and Zhang completely knocked out the open reading frame of the gene at the GFP knock-in site; they still observed that the intensity of the gene-body-localized histone modification H3K79me3 was associated with the expression noise of GFP. This observation suggests the presence of mechanisms in addition to the positive feedback loop between transcription initiation and elongation. In yeast, TSS-proximal nucleosome occupancy regulates the accessibility of the promoter, which influences burst frequency and expression noise [3,6,11,15,17–19]. Because H3K79 methylation regulates the switch between heterochromatin and euchromatin [46], gene-body-localized histone modifications may regulate the accessibility of chromatin in a broader region so that genes localized in this region exhibit reduced gene expression noise [25]. Consistent with this mechanism, Chen and Zhang observed that essential genes tend to be localized in the chromosome regions associated with low expression noise[25], which supported a previous hypothesis that essential genes form clusters in low noise regions to optimize the robustness of transcription [55].

It is also worth noting that although we provided evidence that histone modification can regulate expression noise (Fig **5**), we did not exclude the opposite mechanism that transcriptional bursts may influence the intensities of gene-body-localized histone modifications. The relative contributions of these two mechanisms to the observed correlation between gene-body-localized histone modifications and expression noise deserve further investigations in the future.

### Expansion of the histone code hypothesis

Histone code hypothesis states that the genetic information encoded in DNA is partly regulated by chemical modifications to histone proteins [21]. Although histone modification is tightly associated with transcription, it remains elusive in what specific aspects of transcription do they play a role. For example, both of H3K4me3 and H3K79me2 are associated with active transcription, do they have redundant or separate functions? Here we discovered that burst frequency and burst size, the two independent parameters of transcription, were likely modulated by two distinct groups of histone modifications. Specifically, three gene-body-localized histone markers (H3K36me3, H3K79me2, and H4K20me1) exhibited stronger correlations with burst frequency than with burst size, while one promoter-localized histone marker (H3K4me2) exhibited a stronger correlation with burst size than with burst frequency, in human embryonic cells (Fig **2**). In yeast, two gene-body-localized histone markers (H3K36me3 and H3K79me3) exhibited stronger correlations with burst frequency than with burst size, while three promoter-localized histone markers (H3K4me2, H3K9ac, and H3K14ac) exhibited stronger correlations with burst size than with burst frequency (Fig **4**). Through these histone modifications, the independent regulation of expression level and noise is enabled. Note that additional mechanisms may also be involved in the independent regulation of expression level and noise.

Our finding broadens our understanding of transcription regulation by histone modifications and expands the histone code hypothesis to include the regulation of gene expression noise. Importantly, the evolutionarily conserved patterns in human, mouse, and yeast imply that this epigenetic strategy is probably general and is adopted by many other species.

## Methods and materials

### Estimation of gene expression noise

Single-cell mRNA-seq data in human preimplantation embryos were downloaded from Yan *et al* (GSE36552)[33]. Specifically, the genome-wide expression profiles of 20 single cells from three 8-cell stage embryos were retrieved, which contain 4 cells, 8 cells and 8 cells, respectively. Considering the larger technical error among lowly expressed genes, only genes with expression detected in all 20 cells were used. For each gene, noise (coefficient of variation, CV) was calculated as follows:

$$Noise\left(CV\right)=\frac{\sqrt{\frac{1}{N-3}\sum_{j=1}^{3}\sum_{i=1}^{n_{j}}{\left(x_{ij}-{\bar{x}}_{j}\right)}^{2}}}{\frac{1}{N}\sum_{j=1}^{3}\sum_{i=1}^{n_{j}}x_{ij}}$$ (1)

where *x*_*ij*_ is the expression level of the gene (in the unit of Reads Per Kilobase per Million mapped reads, RPKM) in the *i*^th^ cell of the *j*^th^ embryo. *N* (= 20) is the total number of cells, and *n*_*j*_ is the number of cells in the *j*^th^ embryo. Note that in order to exclude embryo effect in noise estimation, average expression level of each embryo (${\bar{x}}_{j}$) is used in calculating CV. Gene expression noise at the 2/4-cell stage was calculated similarly with Eq (1).

Single-cell mRNA-seq data of the primary outgrowth during human embryonic stem cells (hESC) derivation (passage 0) and hESC of passage 10 were also downloaded from Yan *et al* (GSE36552)[33]. Expression noise is calculated as follows:

$$Noise\left(CV\right)=\frac{\sqrt{\frac{1}{N-2}\sum_{j=1}^{2}\sum_{i=1}^{n_{j}}{\left(x_{ij}-{\bar{x}}_{j}\right)}^{2}}}{\frac{1}{N}\sum_{j=1}^{2}\sum_{i=1}^{n_{j}}x_{ij}}$$ (2)

Eq (2) is similar to Eq (1), except that 3 embryos are replaced with 2 passages.

Single-cell transcriptomes of wild-type mouse embryonic stem cells (mESC) cultured in ground state condition were generated by Kumar *et al* [51]. Expression level and noise (CV) of 94 cells were retrieved from their supplementary data.

Gene expression data at the protein level in single cells were measured with FACS in yeast by Newman *et al* [11]. Expression level and noise (CV) in YPD were retrieved. Newman *et al* also calculated DM (distance of each CV to a running median of CV) to control the negative correlation between expression level and noise[11]. In our study, we separately calculated correlations between histone modifications and expression level or CV. Similar to DM, the difference between the absolute values of those two correlations reflects the deviation from the negative correlation between expression level and CV.

Single cell mRNA-seq data generally have relatively high technical errors, mainly from the variation of single-molecule capture efficiency [36,56]. However, none of known errors generates bias towards certain histone modifications after controlling for gene expression level. In yeast, single-cell protein concentrations were measured by FACS [11], which avoids the high variation of single- molecule capture efficiency in single-cell mRNA-seq.

Total noise includes both extrinsic and intrinsic noise. It was previously reported in yeast that after controlling for cell size, extrinsic noise is significantly reduced. The cell sizes (and cell cycle status—another major source of extrinsic noise) are largely identical in 2-cell and 4-cell human embryos, and we still observed a similar pattern in these stages (Fig **3**). These observations suggest that extrinsic noise is unlikely a major factor influencing our major conclusions of this study.

### Hierarchical clustering

Unsupervised hierarchical clustering analysis was performed on the single-cell transcriptome data from 20 cells with R. To minimize the effect of experimental error in RNA-sequencing on noise calculation, hierarchical clustering analysis was performed on 7741 genes with at least one sequencing read detected in all 20 cells.

### Estimation of burst size and burst frequency from single-cell mRNA-seq data

Based on the estimation that a typical mammalian cell contains 200,000 mRNA molecules [42], we estimated the “amplification factor”, *A*, which equals RPKM_total_/200,000, where RPKM_total_ is the sum of RPKM values of all genes. The number of transcripts for gene *i*, *N*_*i*_, was estimated by RPKM_*i*_/*A*, where RPKM_*i*_ is the RPKM value of gene *i*.

Based on the burst-like model in eukaryotic transcription, burst size can be estimated from the Fano factor (*σ*^2^/*μ*), following previous studies [29],

$$Burst\;size=\sigma^{2}/\mu \text{-}1$$ (3)

where *σ*^2^ and *μ* are the variance and mean of the estimated numbers of transcripts for each gene among cells, respectively. And

$$Burst\;frequency=\mu \times \gamma_{m}/burst\;size$$ (4)

where *γ*_m_ represents mRNA decay rate. Because mRNA decay rate is not available in human embryonic cells, we used two approaches to approximate it. First, we used the average mRNA decay rate estimated from 7 human B-lymphoblastoid cell lines [38]. Second, we assumed that the variance of mRNA decay rates among transcript species was smaller than the variance of mRNA production rates, and used Eq (5) to approximate burst frequency.

Burstfrequency=μ/burstsize (5)

Two approaches led to similar observations (Fig **2C** and **S4** Fig). Genes (*N* = 3350) with estimated burst size > 0.75 were presented in Fig **2C**. The observation kept unchanged when the cutoff of burst size was changed to > 0.5 (*N* = 3733, **S12** Fig) or > 1.0 (*N* = 3057, **S12** Fig).

### Estimation of burst size and burst frequency from FACS data in yeast

Burst size and burst frequency in yeast were estimated with the following equations.

$$Burst\;size=\sigma^{2}/\mu \text{-}1$$ (6)

$$Burst\;frequency=\mu \times \gamma_{p}/burst\;size$$ (7)

where variance (*σ*^2^) and average expression level (*μ*) were retrieved from a previous study [11] and were normalized by the total number of proteins in a yeast cell (~5*×*10^7^) [57]. Protein degradation rate (*γ*_*p*_) was retrieved from a previous study that measured protein degradation rates based on stable isotope labeling with amino acids in cell culture (SILAC) [58]. This degradation rate dataset was used because protein synthesis inhibitors, which perturb normal cell status, were not added to the culture media in this study. Note that the burst size in Eqs 6 and 7 refers to the number of proteins synthesized in each burst (*Burst size*_*p*_) whereas the burst size in Eqs 3–5 refers to the number of mRNAs synthesized in each burst (*Burst size*_*m*_). They are related by *Burst size*_*p*_ = *Burst size*_*m*_ × *k*_*p*_ / *γ*_*m*_, where *k*_*p*_ is the protein translation rate and *γ*_*m*_ is the mRNA degradation rate. The underlying assumption in Eqs 6 and 7 is that the mRNA degradation rate is much higher than the protein degradation rate [41]. We retrieved the half-life data from previous studies [58,59], and found that this was indeed the case (the median mRNA half-life is ~ 7 minutes, while the median protein half-life is ~521 minutes).

### ChIP-seq and ChIP-chip data of histone modifications

Histone modification data in human and mouse were downloaded from the encyclopedia of DNA elements (ENCODE) project [43], in which chromatin immunoprecipitation coupled with high throughput sequencing (ChIP-seq) experiments were performed to measure intensities of histone modifications. All Eight ChIP-seq datasets of euchromatic histone modifications in H1-hESC (BROADPEAK files) were retrieved, under GEO accession numbers GSM733782 (H3K4me1), GSM733670 (H3K4me2), GSM733657 (H3K4me3), GSM733773 (H3K9ac), GSM733718 (H3K27ac), GSM733725 (H3K36me3), GSM1003547 (H3K79me2), and GSM733687 (H4K20me1).

Five ChIP-seq datasets of euchromatic histone modifications in mouse embryonic stem cell line ES-Bruce4 (BROADPEAK files) were retrieved, under GEO accession numbers GSM769009 (H3K4me1), GSM769008 (H3K4me3), GSM1000127 (H3K9ac), GSM1000099 (H3K27ac), and GSM1000109 (H3K36me3). Because H3K79me2 data are not available in any mouse embryonic stem cells, H3K79me2 in cell line CH12 was used instead.

The average intensity of histone modification on each gene was calculated as follows:

$$\frac{1}{L_{G}}\sum_{i=1}^{k}L_{i}\times E_{i}$$ (8)

where *L*_*G*_ is gene length, which is defined as the distance between 2 kilo base pairs (kb) upstream of TSS and TES. *k* is the total peak number of a specific histone modification on a gene. *L*_*i*_ and *E*_*i*_ are the length and the average intensity of the *i*^th^ histone modification peak on the gene, respectively. Since this equation could lead to inaccurate estimation of the intensities of histone modifications that only localize in part of a gene, we also calculated the intensity of histone markers in a modified equation:

$$\frac{1}{L_{P}}\sum_{i=1}^{k}L_{i}\times E_{i}$$ (9)

where gene length (*L*_*G*_) was replaced by the length of genome that cover all peaks on a gene (*L*_*P*_). Two equations result in similar observations (Fig **3A** and **S9C** Fig). Thus, Eq (8) was used in the rest of this study.

Histone modification data in yeast were retrieved from a previous study [47]. In the study, chromatin immunoprecipitation coupled with DNA microarrays (ChIP-chip experiments) were performed for H3K9ac, H3K14ac, H3K4me1, H3K4me2, H3K4me3, H3K36me3, and H3K79me3. Average histone modification intensity from multiple probes was calculated for each gene.

### Permutation test in detecting the difference between *r*_*BF*_ and *r*_*BS*_

To determine if the difference between *r*_*BF*_ and *r*_*BS*_ is significant, we performed permutation by shuffling genes for 1000 times, obtained 1000 *r*_*BF*_ and *r*_*BS*_, and then calculated two-tailed *P* values.

### TATA-box and nucleosome occupancy

TATA-box classification information in human was downloaded from Jin *et al* [52] and Yang *et al* [53]. In Jin *et al*, promoters were further classified into two categories, based on the presence of the canonical TATA-box (TATATAA).

Nucleosome protected DNA sequences in human were retrieved from Gaffney *et al* [54], where high throughput sequencing data were generated from micrococcal nuclease-digested chromatin (MNase-seq). This study contains the highest-resolution map of nucleosome occupancy to date in human. Nucleosome occupancy was calculated in the region between 250/200/150/100 base pairs (bp) upstream of the transcriptional starting site (TSS):

$${\text{Nucleosome occupancy}}_{\text{region length}}=\sum_{i=1}^{\text{region length}}x_{i}$$ (10)

where *x*_*i*_ is the number of reads whose midpoints are *i* bases upstream of TSS.

TATA box-containing genes in yeast were identified by Basehoar *et al* [60]. Genes with occupied proximal-nucleosome (OPN) and depleted proximal-nucleosome (DPN) were identified from the promoter nucleosome occupancy data in yeast [61] by Tirosh and Barkai [19].

### Essential genes and genes encoding protein complex subunits

Human orthologs of mouse essential genes were identified by Georgi *et al* [62], and were defined as essential genes in human in our study. Genes encoding protein complex subunits were retrieved from CORUM (http://mips.helmholtz-muenchen.de/genre/proj/corum) and Havugimana *et al* [63].

### Estimation of technical noise in human embryonic cells

Assuming that technical-error-derived mRNA molecule number follows a Poisson distribution, we can deduce that the line corresponding to technical noise has a slope of -0.5 and an intercept of 1/2×log_2_(*A*), or 0.73 (Fig **1B**), where *A* is the amplification factor estimated above. Considering that human embryonic cells are larger than average human cells and thus have more mRNA molecules than 200,000, the actual technical noise should be lower than estimated here.

### KEGG enrichment analysis

GOstats [64] was used to calculate the KEGG (Kyoto Encyclopedia of Genes and Genomes) pathways that are enriched in each of the 4 groups defined in Fig **1B**. The background gene set is all genes in the other three groups.

### Calculation of relative H3K79me2/H3K4me3 intensity ratio in Fig 6B and 6C

For each gene in a pathway, we calculated the average H3K79me2 and H3K4me3 intensity as previously described, and then calculated the intensity ratio between them. As a control, for each gene in autophagy or oxidative phosphorylation signaling pathway (Group 1), we identified 100 genes with similar expression levels from Group 2, calculated the H3K79me2/H3K4me3 intensity ratio for each of them, and get the median ratio of these 100 genes. Finally, the intensity ratio of each gene in autophagy or oxidative phosphorylation signaling pathway was divided by the median ratio of these 100 control genes. Relative intensity ratios in Wnt signaling pathway or Jak-STAT signaling pathway (Group 4) were calculated with the same method, only that the control genes were identified from Group 3.

### Construction of yeast strains

Yeast strains *dot1Δ0* and *hoΔ0* were constructed by PCR-mediated gene disruption with an auxotrophic marker gene *URA3*. Briefly, *URA3* sequence was PCR amplified from plasmid pRS416, and the amplicon was transformed into haploid yeast strains BY4741 (*MAT***a** *his3Δ1 leu2Δ0 met15Δ0 ura3Δ0*) and BY4742 (*MATα his3Δ1 leu2Δ0 lys2Δ0 ura3Δ0*), respectively. Yeast cells were selected on synthetic complete medium with uracil dropped-off (SC-uracil); genomic DNA was extracted from colonies and PCR was performed to verify successful gene deletion. *HO* is a site-specific endonuclease required for homothallic switching, and is non-functional in strain *s288c* [65]. Thus, *ho*::*URA3* was used here to control the potential noise effect of the auxotrophic marker *URA3*[66] in *dot1*::*URA3*. The DNA oligos used in this study are listed in **S3** Table.

To estimate protein expression noise in *dot1Δ0* and *hoΔ0* strains, seven genes with high-intensity H3K79 methylation[47] and average protein level larger than 1000 (arbitrary unit) in Newman *et al* [11] were randomly chosen as reporters (**S3** Table). Lowly expressed genes were excluded to ensure accuracy in quantifying florescence intensity. A series of *GFP* strains were generated on the background of BY4741 *ho*::*URA3* and BY4741 *dot1*::*URA3*; in each of them, *GFP* was fused to the C-terminus of a reporter protein, following the protocol at Yeast Resource Center (http://depts.washington.edu/yeastrc/). In brief, GFP-KANMX6 cassette was PCR amplified from plasmid pFA6a-GFP(S65T)-KANMX6, and was transformed into BY4741 *ho*::*URA3* and BY4741 *dot1*::*URA3*, respectively. A series of *dTomato* strains were generated similarly on the background of BY4742 *ho*::*URA3* and BY4742 *dot1*::*URA3*, respectively. *dTomato* sequence and HYGMX4 were first PCR amplified from plasmids pRSET-B *dTomato* and pBS10, respectively, and were seamlessly ligated and cloned into PUC19 by GeneArt Seamless Cloning and Assembly Kit (Life Technology). This dTomato-HYGMX4 cassette was used to fuse *dTomato* to C-terminus of reporter proteins. Double fluorescence diploid strains with homozygous deletion of *DOT1* (*MAT***a**/*MATα dot1*::*URA3*/*dot1*::*URA3 GeneX-GFP-KANMX6/GeneX–dTomato-HYGMX4*; *GeneX* is one of the reporter genes) were obtained by crossing *BY4741 MAT***a** *dot1*::*URA3 GeneX-GFP-KANMX6* with *BY4742 MATα dot1*::*URA3 GeneX–dTomato-HYGMX4* on YPD agar plate, followed by selection on YPD agar plate supplemented by G418 (AMRESCO, 200μg/ml) and hygromycin B (AMRESCO, 300μg/ml). Double fluorescence diploid strains with homozygous deletion of *HO* (*MAT***a**/*MATα ho*::*URA3*/*ho*::*URA3 GeneX-GFP-KANMX6/GeneX–dTomato-HYGMX4*) were generated similarly.

### Single cell preparation in yeast

Yeast cells were cultured in YPD liquid media (1% Yeast extract, 2% Peptone, and 2% Dextrose, mass/volume) and were collected at the mid-log phase. Cells were washed by 1×PBS (137 mM NaCl, 2.7 mM KCl, 10 mM Na_2_HPO_4_, and 1.8 mM KH_2_PO_4_, pH 7.4) and were placed on ice.

### Fluorescence-activated cell scanning

The fluorescence intensities of EGFP and dTomato in single cells were measured with FACSAria III cell sorter (BD Biosciences). GFP was excited by 488nm laser and was detected through 530/30 nm emission filter; dTomato was excited by 561nm laser and was detected through 610/20 nm emission filter. Cells containing single fluorescence protein were also prepared to perform fluorescence compensation in FACS. Three biological replicates were performed for each sample.

Cells were gated by FSC-A, SSC-A, and FSC-W/FSC-H ratio to exclude cells with extraordinary size or complexity, as well as doublets and cell bulks. Then cells with both green and red fluorescence were identified, and were used for subsequent analysis. About 40,000 double-fluorescence events were recorded in each replicate.

Intrinsic noise was calculated following previous studies [1,3].

$$\mu =\sqrt{\bar{g}\bar{r}}$$ (11)

$$Intrinsic\;CV^{2}=\frac{\sigma^{2}}{\mu^{2}}=\frac{\bar{{\left(g-r\right)}^{2}}}{2\bar{g}\bar{r}}$$ (12)

where *g* and *r* are the normalized EGFP intensity and dTomato intensity, respectively, in single cells.

Burst frequency was estimated based on gamma distribution [67]. The intensity of EGFP fluorescence was normalized with the dTomato intensity of the same cell and single-cell fluorescence intensity of normalized EGFP was fit to a gamma distribution, in which process rate parameter (*β*) and shape parameter (*α*) were estimated. Burst frequency was calculated with the following equation.

Burstfrequency=α (13)

### Microscopy

Yeast cells were grown to mid-log phase and washed once by 1×PBS (pH 7.4). Fluorescent images were acquired using confocal microscope (DIGITAL ECLIPSE C1Si, Nikon, Japan) equipped with 488 nm and 543 nm lasers, under an oil-immersed objective at ×100 magnification.

### Western blot assay

Yeast cells were cultured in YPD liquid media (1% Yeast extract, 2% Peptone, and 2% Dextrose, mass/volume), which were collected at log phase (50 ml, OD_660_ around 0.7), washed, and immediately suspended in 400μl lysis buffer (50 mM HEPES-KOH, pH 7.5, 140 mM NaCl, 1 mM EDTA, 1% Triton X-100, 0.1% sodium deoxycholate, and Protease Inhibitor Cocktail). The suspension was mixed with glass beads, vortexed for 10 min at 4°C, and sonicated without beads for 50 times on ice (10-sec pulse at 195W followed by 20-sec rest). After 20-minute centrifugation at 10,000g, the supernatant was transferred to a new tube and boiled for 5 minutes in SDS sample buffer. After centrifugation (14,000g for 5 minutes), 40μl of the resulting lysates was subjected to western blot assay. Anti-H3K79 antibody, Anti-H3 antibody, and Anti-GAPDH antibody were purchased from Abcam (ab3594, ab1791 and ab9484, respectively). H3 and GAPDH were used as loading controls. The anti-H3K79 antibody (ab3594) binds to all three H3K79 methylations. Primary antibodies were used at 1:8000 dilution. Horse radish peroxidase (HRP)-conjugated secondary antibodies were purchased from Cell Signaling Technology (Cat. # 7074, 1:5,000 dilution; # 7076, 1:10,000 dilution).

## Supporting information

S1 Fig Essential genes (**A**) and genes encoding protein complex subunits (**B**) are enriched in the high-expression-low-noise group (group 2). Hypergeometric test was used to determine the enrichment significances.
(EPS)

S2 Fig mRNA decay rate is weekly correlated with expression level (**A**) and noise (**B**) in human embryonic cells. Spearman’s correlation coefficients and corresponding *P* values are shown.
(EPS)

S3 Fig Schematic diagram of correlations between expression level/noise and burst size/burst frequency according to the burst-like gene expression model. Expression level is determined by both burst size and burst frequency (**A-B**), while noise is determined only by burst frequency (**C-D**).
(EPS)

S4 Fig After considering mRNA decay rate in the estimation of burst parameters, the pattern in Fig 2 persisted. (EPS)

S5 Fig Histone modification conservation between cell lines hESC and GM12878. Average modification intensity was calculated on each gene for each histone marker. Then genes were divided into 10 equal-sized bins according to modification intensity. Spearman correlation coefficients were calculated based on raw data.
(EPS)

S6 Fig The correlations between the intensities of histone modifications in GM12878 and the expression level (noise) in cells of the 8-cell stage embryos. The pattern observed in Fig 2 is largely unchanged.
(EPS)

S7 Fig Genes in the oxidative phosphorylation signaling pathway have lower relative H3K79me2/H3K4me3 intensity ratios. Genes in the Jak-STAT signaling pathway have higher relative H3K79me2/H3K4me3 intensity ratios. Grey boxes indicate no available intensity ratio data. Similar to Fig 6.
(EPS)

S8 Fig Usage preference of histone modifications among genes with different expression level and noise in yeast. **A**, Genes are divided by the major axis and minor axis into 4 groups based on expression level and noise. **B**, Enrichment of histone modifications in 4 groups of genes. Hypergeometric test was used to determine the enrichment significances.
(EPS)

S9 Fig “Division of labor” among histone modifications is also observed in human embryonic stem cells (**A**), after excluding human homologs of the bimodally expressed genes in mouse at 2/4-cell stage (**B**), when calculating histone modification intensities only in ChIP-Seq peak-regions (**C**), and in mouse embryonic stem cells (**D**).
(EPS)

S10 Fig The difference in the intensity of H3K79me2 of orthologous genes between human and mouse can predict the evolutionary divergence of expression noise but not expression level. (EPS)

S11 Fig TATA box and nucleosome occupancy are not correlated with noise in human embryo cells. **A**, No significant difference in noise was detected between TATA-containing and TATA-less genes. *t* test was used to calculate the significance. **B**, No significant correlations between noise and promoter nucleosome occupancy were detected in all 4 ranges.
(EPS)

S12 Fig Different burst size cutoffs were used and the pattern observed in Fig 2 is largely unchanged. (EPS)

S1 Table KEGG terms enriched in each group in Fig 1B. (PDF)

S2 Table KEGG terms enriched in each group (95% confidence intervals). Similar to S1 Table, except that genes with significant deviations from the 95% confidence intervals of the major axis and minor axis were divided into 4 groups.
(PDF)

S3 Table Primers used in this study. (PDF)
