# Supplementary material for: Independent regulation of gene expression level and noise by histone modifications
Source: PLoS Comput Biol. 2017 Jun 30;13(6):e1005585. doi: 10.1371/journal.pcbi.1005585 (PMC5513504; doi:10.1371/journal.pcbi.1005585)
Supplement: S1 Table — (PDF) [file pcbi.1005585.s013.pdf]

S1 Table. KEGG terms enriched in each group in Fig **1B**.

Group 1			
**KEGG ID**	*P*-value*	Odds Ratio	KEGG Term
**3010**	0.00	7.51	Ribosome
**3008**	0.00	4.38	Ribosome biogenesis in eukaryotes
**190**	0.00	2.73	Oxidative phosphorylation
**3013**	0.00	2.07	RNA transport
**3050**	0.00	4.00	Proteasome
**5012**	0.00	2.30	Parkinson's disease
**5010**	0.00	2.30	Alzheimer's disease
**3040**	0.00	2.10	Spliceosome
**5016**	0.00	1.78	Huntington's disease
**4260**	0.00	3.06	Cardiac muscle contraction
**270**	0.00	3.68	Cysteine and methionine metabolism
**480**	0.00	3.96	Glutathione metabolism
**1230**	0.01	2.55	Biosynthesis of amino acids
**4978**	0.01	3.22	Mineral absorption
**5322**	0.01	2.08	Systemic lupus erythematosus
**4932**	0.01	1.66	Non-alcoholic fatty liver disease (NAFLD)
**4140**	0.01	2.97	Regulation of autophagy
**1200**	0.02	1.91	Carbon metabolism
**330**	0.02	2.63	Arginine and proline metabolism
**4145**	0.03	1.66	Phagosome
**3060**	0.04	2.41	Protein export
Group 2			
**3018**	0.00	3.12	RNA degradation
**3040**	0.00	2.15	Spliceosome
**3015**	0.00	2.35	mRNA surveillance pathway
**3020**	0.01	2.73	RNA polymerase
**3013**	0.01	1.60	RNA transport
**900**	0.02	3.37	Terpenoid backbone biosynthesis
**240**	0.03	1.80	Pyrimidine metabolism
**4141**	0.03	1.51	Protein processing in endoplasmic reticulum
**4530**	0.04	1.72	Tight junction
**4919**	0.04	1.68	Thyroid hormone signalling pathway
**5100**	0.05	1.81	Bacterial invasion of epithelial cells
Group 3			
**5231**	0.00	3.51	Choline metabolism in cancer
**4070**	0.00	3.51	Phosphatidylinositol signalling system
**4650**	0.00	3.42	Natural killer cell mediated cytotoxicity
**4662**	0.00	3.42	B cell receptor signalling pathway
**4930**	0.00	6.36	Type II diabetes mellitus
**4973**	0.00	6.36	Carbohydrate digestion and absorption
**5160**	0.00	2.80	Hepatitis C
**5223**	0.00	3.92	Non-small cell lung cancer
**561**	0.00	4.71	Glycerolipid metabolism
**4015**	0.00	2.19	Rap1 signalling pathway

**5162**	0.00	2.62	Measles
**4725**	0.00	2.73	Cholinergic synapse
**5205**	0.00	2.07	Proteoglycans in cancer
**5230**	0.00	2.98	Central carbon metabolism in cancer
**5218**	0.00	3.59	Melanoma
**1100**	0.00	1.41	Metabolic pathways
**5213**	0.00	3.07	Endometrial cancer
**5214**	0.00	3.07	Glioma
**5221**	0.00	3.18	Acute myeloid leukaemia
**4664**	0.00	3.18	Fc epsilon RI signalling pathway
**531**	0.00	10.53	Glycosaminoglycan degradation
**4150**	0.00	2.66	mTOR signalling pathway
**4012**	0.00	2.66	ErbB signalling pathway
**4380**	0.00	2.52	Osteoclast differentiation
**4919**	0.00	2.14	Thyroid hormone signalling pathway
**4370**	0.00	3.00	VEGF signalling pathway
**5200**	0.01	1.66	Pathways in cancer
**5144**	0.01	16.83	Malaria
**4014**	0.01	1.92	Ras signalling pathway
**4940**	0.01	Inf	Type I diabetes mellitus
**4210**	0.01	2.63	Apoptosis
**4660**	0.01	2.36	T cell receptor signalling pathway
**4666**	0.01	2.27	Fc gamma R-mediated phagocytosis
**4611**	0.01	2.19	Platelet activation
**5152**	0.01	2.03	Tuberculosis
**4810**	0.01	1.79	Regulation of actin cytoskeleton
**564**	0.01	2.38	Glycerophospholipid metabolism
**4917**	0.01	2.74	Prolactin signalling pathway
**5215**	0.01	2.27	Prostate cancer
**4960**	0.01	3.38	Aldosterone-regulated sodium reabsorption
**4062**	0.01	1.90	Chemokine signalling pathway
**5164**	0.01	1.94	Influenza A
**4142**	0.01	1.94	Lysosome
**4066**	0.01	2.06	HIF-1 signalling pathway
**4071**	0.01	2.01	Sphingolipid signalling pathway
**4720**	0.01	2.59	Long-term potentiation
**562**	0.01	2.59	Inositol phosphate metabolism
**4022**	0.02	1.89	cGMP-PKG signalling pathway
**5142**	0.02	2.20	Chagas disease (American trypanosomiasis)
**4921**	0.02	1.92	Oxytocin signalling pathway
**4750**	0.02	2.64	Inflammatory mediator regulation of TRP channels
**4914**	0.02	1.91	Progesterone-mediated oocyte maturation
**5212**	0.02	2.49	Pancreatic cancer
**4923**	0.03	2.81	Regulation of lipolysis in adipocytes
**4144**	0.03	1.59	Endocytosis
**4723**	0.03	2.53	Retrograde endocannabinoid signalling
**4620**	0.03	2.35	Toll-like receptor signalling pathway
**4640**	0.04	3.16	Hematopoietic cell lineage
**4974**	0.04	3.16	Protein digestion and absorption
**5033**	0.04	Inf	Nicotine addiction
**5145**	0.04	1.96	Toxoplasmosis

**4510**	0.04	1.64	Focal adhesion
**5222**	0.04	2.03	Small cell lung cancer
**4010**	0.04	1.55	MAPK signalling pathway
**4550**	0.04	1.73	Signalling pathways regulating pluripotency of stem cells
**5220**	0.05	2.02	Chronic myeloid leukaemia
**4722**	0.05	1.71	Neurotrophin signalling pathway
**1210**	0.05	4.20	2-Oxocarboxylic acid metabolism
Group 4			
**4060**	0.00	4.22	Cytokine-cytokine receptor interaction
**4630**	0.00	3.41	Jak-STAT signalling pathway
**4080**	0.00	5.09	Neuroactive ligand-receptor interaction
**980**	0.00	7.57	Metabolism of xenobiotics by cytochrome P450
**5321**	0.00	9.92	Inflammatory bowel disease (IBD)
**310**	0.00	3.70	Lysine degradation
**4550**	0.00	2.21	Signalling pathways regulating pluripotency of stem cells
**2010**	0.00	5.67	ABC transporters
**4913**	0.00	4.06	Ovarian steroidogenesis
**534**	0.00	Inf	Glycosaminoglycan biosynthesis - heparan sulfate
**4020**	0.01	2.28	Calcium signalling pathway
**5217**	0.01	4.96	Basal cell carcinoma
**140**	0.02	7.07	Steroid hormone biosynthesis
**982**	0.02	7.07	Drug metabolism - cytochrome P450
**5166**	0.02	1.60	HTLV-I infection
**5204**	0.02	3.96	Chemical carcinogenesis
**5150**	0.02	11.30	Staphylococcus aureus infection
**4350**	0.03	2.03	TGF-beta signalling pathway
**4724**	0.03	2.09	Glutamatergic synapse
**450**	0.03	4.71	Selenocompound metabolism
**4916**	0.04	1.94	Melanogenesis
**4912**	0.04	1.99	GnRH signalling pathway
**4514**	0.04	2.23	Cell adhesion molecules (CAMs)
**5032**	0.04	2.13	Morphine addiction
**4610**	0.04	5.65	Complement and coagulation cascades

**P* values were calculated with hypergeometric test. The background gene set in the enrichment analysis is all genes in the three other groups.
