# Supplementary material for: Independent regulation of gene expression level and noise by histone modifications
Source: PLoS Comput Biol. 2017 Jun 30;13(6):e1005585. doi: 10.1371/journal.pcbi.1005585 (PMC5513504; doi:10.1371/journal.pcbi.1005585)
Supplement: S3 Table — (PDF) [file pcbi.1005585.s015.pdf]

S3 Table. Primers used in this study.

Primer sequence (5'--3')	Primer name
**Primers for generating *dotΔ0* and *hoΔ0* strains**	
**CATCAAGGAGGTCACCAGTAATTGTGCGCTTTGGT TACATTTTGTTGTACAGTAagcttttcaattcaattcatcattt**	YDR440W-URA3-del-F
**ATGTTATTTCTACTTAGTTATTCATACTCATCGTTAA AAGCCGTTCAAAGTGCCgggtaataactgatataattaaatt**	YDR440W-URA3-del-R
**CATCAAGGAGGTCACCAGTA**	YDR440W-del-F-verify
**ATGTTATTTCTACTTAGTTA**	YDR440W-del-R-verify
**TAGCTCTAAATCCATATCCTCATAAGCAGCAATCAA TTCTATCTATACTTTAAAagcttttcaattcaattcatcattt**	YDL227C-URA3-del-F
**AAATATTAAATTTTACTTTTATTACATACAACTTTTT AAACTAATATACACATTgggtaataactgatataattaaatt**	YDL227C-URA3-del-F
**TAGCTCTAAATCCATATCCTCATAA**	YDL227C-del-F-verify
**AAATATTAAATTTTACTTTTATTACATAC**	YDL227C-del-R-verify
**Primers for dTomato-Hyromycin B seamless cloning**	
**ATTCGAGCTCGGTACGGTCGACGGATCCCCGGGTTAATT AACAGTgtgagcaagggcgaggaggtc**	dTomato-F
**GGCGCGCC TTACTTGTACAGCTC**	dTomato-R
**ACAAGTAAggcgcgccagatctg**	HygroB-F
**GCCAAGCTTGCATGCCATCGATGAATTCGAGCTCG**	HygroB-R
**Primers for fusing fluorescent proteins to the C-terminus of noise reporter genes**	
**CAAGTTGCTAGATCCGATTGGCTGCTATTGAAGAAGCTG AAGGTCGTTTTCGGCATCGAAggtcgacggatccccggg**	YAL035W-F
**TATTTAATTCATTAAATTTTGTAGAATGTGATTGGGTTGA CAAGTCAGCGTATGCCATGCatcgatgaattcgagctcg**	YAL035W-R
**CAAGTTGCTAGATCCGATTG**	YAL035W-F-verify
**TATTTAATTCATTAAATTTT**	YAL035W-R-verify
**GAATTGAGAAAGGCTGAAGTTGGTTTGAAGAGAGTTGTC ACCAAGGCCATGTCTTCTCGTggtcgacggatccccggg**	YDL229W-F
**GAGAAAACAAAATTTATATACAATATAAGTAATATTCATA TATATGTGATGAATGCAGTCatcgatgaattcgagctcg**	YDL229W-R
**GAATTGAGAAAGGCTGAAGT**	YDL229W-F-verify
**CATCTCAAGATACAAAAAGC**	YDL229W-R-verify
**GAAGTTTTGAAACAGTTGCGTAACTTGAAAGATGAAAAG AGAGGTGACAACGTCTTAGCTggtcgacggatccccggg**	YIL078W-F
**CATTTTATTCTTTACAAAACATAAATTAAATAAGTCAAAA TTTAACTACATAAAATCTCAatcgatgaattcgagctcg**	YIL078W-R
**GAAGTTTTGAAACAGTTGCG**	YIL078W-F-verify
**ATATAGGTATATGTTTGGGCCAA**	YIL078W-R-verify
**GTTCATGGTAAAGGTGAAGACAGCTTATTATGCTCTGAT CTGGTGAATGGTCTAATGCAGggtcgacggatccccggg**	YNL287W-F
**CATTTCTTTTAATACTATTGATAACAAAAGAAGGGATTAA CTGAAATAAAATTTCACAGCatcgatgaattcgagctcg**	YNL287W-R
**GTTCATGGTAAAGGTGAAGA**	YNL287W-F-verify
**TTTCTTCCATTTCGTTTTCT**	YNL287W-R-verify
**TCATCCATTCATTGGAGATTCCAAAGAGAGTTGTGGGAC GAAGAAAAGGAAATTGTTTTGggtcgacggatccccggg**	YPL145C-F

**GAAGAAAAGATAGATAATATATTAGTGCAACGGTAACAA GTTGTTACTTTATCGTTCTCCatcgatgaattcgagctcg**	YPL145C-R
**TCATCCATTCATTGGAGATT**	YPL145C-F-verify
**AAGGTTTTTTCTGCTTCATC**	YPL145C-R-verify
**GTTGACAAGACTGAAAAGGCCGCTAAGGTTACCAAGGCT GCTCAAAAGGCTGCTAAGAAAggtcgacggatccccggg**	YPR080W-F
**AACTAATAGATAAGATTTAAATATAAAAGATATGCAACTA GAAAAGTCTTATCAATCTCCatcgatgaattcgagctcg**	YPR080W-R
**GTTGACAAGACTGAAAAGGC**	YPR080W-F-verify
**AACTAATAGATAAGATTTAA**	YPR080W-R-verify
**GATAAAACTGCTCAATTGACTGTTGAAGATGGTGACAAT TGGGAAGTTGTTGGTAAGAAAggtcgacggatccccggg**	YPR163C-F
**ACAAGAAAGAAAAAAAGCAAACAGTAAATACAAATGTAC ATTTTATCATCATACAACACTatcgatgaattcgagctcg**	YPR163C-R
**GATAAAACTGCTCAATTGAC**	YPR163C-F-verify
**AATGCTTTTTCTTAGAACTT**	YPR163C-R-verify
